# Diagnostic Utility of Combined CEA, CA15-3 and CA125 Biomarkers and Cytomorphology in Suspicious and Malignant Serosal Fluid

**DOI:** 10.30699/IJP.2021.130458.2450

**Published:** 2021-05-09

**Authors:** Zahra Rahemi, Abdolreza Javadi, Behrang Kazeminejad, Abdolali Ebrahimi, Houman Vosough, Afsoon Taghavi, Shahriar Dabiri

**Affiliations:** 1 *Department of Pathology, Imam Hossein Hospital, Shahid Beheshti University of Medical Sciences, Tehran, Iran*; 2 *Imam Hossein Central Medical Laboratory, Shahid Beheshti University of Medical Sciences, Tehran, Iran*; 3 *Department of Pathology, Modarres Hospital, Shahid Beheshti University of Medical Sciences, Tehran, Iran*; 4 *Department of Pathology, Shahid Beheshti University of Medical Sciences, Tehran, Iran*; 5 *Department of Pathology, Afzalipour Medical School, Kerman, Iran*

**Keywords:** Body fluids, CA125, CA15-3, CEA, Chemiluminescence

## Abstract

**Background & Objective:**

Early detection of malignancies in the serous fluids has been remained an issue. A classic diagnostic tool for the ascites and pleural effusions is cytologic study (morphology) with approximately 98% specificity for the detection of cancer cells. This study aimed to evaluate the diagnostic value of three complementary markers in the serosal fluids of patients with malignant cytology and suspected cases.

**Methods:**

Seventy two patients with serosal effusion treated in three teaching hospitals were studied. The cases underwent a diagnostic workup to determine the pleural effusion malignancy and etiologies. Complementary markers, including CEA, CA15-3, and CA125 were measured in serosal fluids of three categories of benign, suspicious, and malignant. The study was carried out by Chemiluminescence immunoalayzer. The morphologies were re-evaluated by a consulting Cytopathologist.

**Results:**

Of 72 serosal fluid specimens, 41 (56.9%) were related to pleural effusion and 31 (43.1%) were related to ascites. The sensitivity of CEA, CA125, and CA15-3 biomarkers were 64, 84, and 68%, respectively, and the specificity of each test was 100, 86, and 96%, respectively. This was statistically achieved for the combination of the area of markers below the curve (AUC), 0.93 and 90% sensitivity and 91% specificity.

**Conclusion:**

The results suggest that complementary CA125, CA15-3, and CEA markers assayed with well-developed immunoassay method might be useful in the differentiation between malignant and benign effusions while combined with conventional cytology. CA125 yielded a significant correlation between cytomorphology and biomarkers based on the correlation coefficient analysis.

## Introduction

Serosal effusion is one of the complications of a large number of diseases (1). The presence of serous fluid effusion, including pleural effusion and ascites, is a common and challenging diagnostic issue caused by different with etiologies varied form neoplastic such as metastatic involvement to non-neoplastic of infectious and non-infectious agents. The most important goal of choosing the treatment strategies is to differentiate the causes of malignant from non-malignant effusions (1). Many of the existing diagnostic methods are inadequate and invasive. Initial diagnosis is based on the thoraco-abdominal centesis, cytology, cell blocking, and biochemical assay of fluids (2). One of the most common diagnostic tools for the serous fluids is the detection of malignant cells using conventional cytology, which has a specificity of about 98% but a maximum sensitivity of 60% (3). In a study, cytology showed a clinical sensitivity of 57% and specificity of 89% for the detection of malignant cells in the effusion samples (4). Different studies and meta-analysis on the patients with unknown origin serosal effusions reported diagnostic accuracy of carcinoma embryonic antigen (CEA), CA15-3, and CA19-9. The results highlighted that applying one biomarker alone for the malignant effusion cannot be advised. The definite role of the combined tumor markers by conventional immunoassay has been reported controversial (5-8). 

On the other hand, a systemic review of 21 articles indicated diagnostic value of combination of CA153, CA199 and CYFRA-21-1 in pleural effusions (7), and also emphasized on utility of CA15-3 for diagnosis of malignant pleural effusions (9). Moreover, elevated levels of both CEA and CA19-9 in peritoneal cavity (11) and peritoneal washing fluid in gastric and pancreatic carcinoma cases undergoing surgical treatment might signalize advanced stage of the illness (12).

A combination of complementary tumor markers, including CEA, CA125, and CA15-3 with a defined diagnostic cut-off point can be used as an adjunct to diagnose suspicious patients in cytology, confirming malignant and benign groups, and accelerate the decision-making process. To select multiple tumor markers, only complementary tumor-specific markers should be used (13).

Based on the updated systematic reviews, the present study used most reliable tumor markers from multiple epitopes of specific tumor antigens in serous fluids, applying finest automated Chemiluminescence immunoassay. This method is a non-invasive method and can initially replace invasive procedures, such as thoracoscopy and laparoscopic biopsy with a clinical sensitivity of about 95%. In this immunoassay method, the protein antigen is labeled with fluorescein and then reacted with specific antibody to the antigen (13).

In this study, diagnostic value indices, including sensitivity, specificity, as well as predictive values of tumor markers alone and in combination are evaluated in these patient categories.

## Material and Methods

The study was conducted on patients with invasive carcinoma of the breast who had undergone surgery between the years 2010 and 2015 in Rohani Hospital, Babol, Iran. All the formalin-fixed paraffin blocks of the tumors were available. The inclusion criteria were presence of an invasive carcinoma component in the available paraffin block along with normal breast tissue in the tumor margin. Those cases diagnosed as carcinoma in situ or presented with extensive tumor necrosis in all slides were excluded from the study. Clinicopathological data, including age, gender, tumor stage, and grade, were retrieved from standard reports, which were prepared according to the American Joint Committee on Cancer (AJCC), 7^th^ edition.

**Immunohistochemistry**

To highlight lymphatic and blood vessels, two 3-4 µm thick sections were prepared from each block (one block per case), and IHC staining for D2-40 and CD31 (Dako, Glostrüp, Denmark) was performed. Sections were dewaxed at 60°C in an oven for about one hour, and then they were put in xylol and rehydrated through a descending concentration of ethanol. For antigen retrieval, sections were microwaved for 15 minutes in ethylenediaminetetraacetic acid (EDTA) buffer (pH=9). Sections were left at room temperature for 15 minutes to cool down. They were washed in tris-buffered saline (TBS) for five minutes and incubated in 3% H_2_O_2_ in dark humid condition. After that, they were washed in TBS for five minutes. Sections were incubated with primary antibody for 60 minutes at room temperature and with secondary antibody for 30 minutes. Sites of binding were detected by a 10-minute incubation with diaminobenzidine (DAB). 

IHC and archived H&E slides were reviewed by two pathologists, not knowing the pathology report. Data were imported in SPSS 20 (SPSS Inc., Chicago, Ill., USA); Chi-square, Fisher’s exact, and *t* tests were performed for statistical analysis.

## Results

The age range of the patients was 32 to 94 years old. Participating patients were assigned into three benign, malignant, and suspicious cytomorphology groups. There were 12 males and 10 females with the mean age of 73.73 ± 13.9 years in benign cytology group, 9 males and 22 females with the mean age of 59 ± 13.34 years in malignant cytology group, and 5 males and 14 females with the mean age of 59.84 ± 15.71 years in suspicious cytology group. The age range in the benign, malignant, and suspicious cytology groups was 43-94, 35-83, and 32-84 years, respectively. Although the age did not represent a clinically significant difference between the categories, it was statistically significant (*P*=0.001), so that the lowest and the highest age range belonged to the patients in the malignant and benign cytology groups, respectively (Table 1).

Of 72 serosal fluids, 41 (56.9%) were related to pleural effusion and 31 (43.1%) were related to peritoneal effusions. Morphologically, 31 samples (43.4%), 22 samples (30.6%), and 19 samples (26.4%) were allocated in the benign, malignant, and suspicious categories, respectively. Three groups were matched in terms of sex, and there was no statistically significant difference between the above groups in this regard (*P*=0.103).The mean levels of tumor markers in all cytology groups are shown in Table 2. The mean levels of CA15-3, CA125, and CEA tumor markers were 69.91 ± 93.88, 39 ± 395.44, and 157.17 ± 234.13, respectively. The highest CA125, CEA, and CA15-3 levels were observed in the malignant cytology group, which was significantly different from the negative cytology group. Indeed, all markers exhibited a similar pattern in all groups. The mean CA15-3 levels in the malignant and benign cytology groups were 110.02 ± 105.83 and 11.79 ± 9.62, respectively, being statistically significant (*P*<0.001).

The mean CA125 levels in the malignant and negative cytology groups were 456.47 ± 121.15 and 286.25±166, respectively, being statistically significant (*P*<0.001). We observed that the mean level of the above tumor markers in the suspicious cytology group was 422.32 ± 136.43. There was a statistically significant difference between benign and suspicious cytology groups (*P*=0.008). The analysis showed a mean level of CEA at 256.71±260.38 and 26.6± 116.191 in the cytology malignant and benign groups, respectively, which was considered statistically significant (*P*=0.001) (Table 2)..

**Table 1:** Age and sex distribution of patients (Mean ± SD).

		Cytomorphology.				
	Total	Benign	Malignant	Suspicious	P-value	Pairwise comparison
Mean ± SD	63.72±15.48	73.73±13.9	59±13.34	59.84±19.71	0.001**	[p(1,2)=0.001], [p(1,3)=0.0080]
Median	34(32,94)	77(43,94)	58(35,83)	62(32,84)	0.103*	
Male	26(36.1%)	12(54.1%)	9(29%	5(26.3%)		
Female	46(63.9%)	10(45.5%)	22(71%)	14(73.7%)		

*:P-value is based on the chi-square test.

**:P-value is based on the ANOVA (multiple comparison corrections were carried out using the Bonferroni method in all the above analyses).

**Table 2:** Serosal fluid levels of CA15-3, CA 125, and CEA in three cytology groups (Mean ± SEM).

Tumor markers		Cytomorphology				
	Total	Benign (n=31)	Malignant (n=22)	Suspicious (n=19)	P- value	Pairwise comparison
CA15-3 (U/mL)**	69.91±93.88 26.05(1.2, 339.6)	11.79±9.62 8.8(1.2, 34.5)	110.02±105.83 84.4(2.5,300)	71.77±9531 26.8(5.3,339.6)	>0.001	[p(1,2)=<0.001]
CA125 (U/mL)	396.44±156.79 500(3.8,500)	286.25±166 235.85(3.8,500)	456.47±121.15 500(21.9,500)	422.32±136.43 500(31.1,500)	>0.001	[p(1,2)=<0.001], [p(1,2)=0.008],
CEA (ng/mL)	157.17±234.13 5.1(0.2,550	26.6±116.91 1.35(0.2,550)	256.71±260.38 152.4(0.2,550)	145.97±221.91 9.4(0.3,550)	0.001	[p(1,2)=0.001]

*:P-value is based on the ANOVA (multiple comparison corrections were carried out using Bonferroni method in the above analyses).

**:Units are based on the ADVIA Centaur® assay chart (Version 1.0.CU Test Definitions,078D0022-22 Rev. A, 2010-03).

The Spearman correlation coefficient was cal-culated to investigate the association between tumor markers and cytological results. As mentioned earlier, the highest CA15, CEA, and CA15-3 levels were observed in the malignant cytology group, as presented in the above Table. In other words, the correlation coefficient of all three tumor markers revealed that the cytological results were more likely to be malignant with increasing tumor marker levels, CA125 showed higher correlation coefficient than the other two tumor markers (r=0.345, *P*=0.003) (Table 3).

The etiologies of cytologically malignant effusions and the sites of origin are demonstrated in Table 4.

**Table 3:** Correlation coefficients and relationships between tumor markers with cytomorphological results

	CA15-3	CA125	CEA
Cytology Spearman correlation coefficient	0.26*	0.345**	0.311**
P-value	0.028	0.003	0.075

**Table 4:** Distribution of Malignant Serosal fluids by confirmed etiology

Causes	n
Malignant	31
Secondary to adenocarcinoma	
Lung	6
Breast	7
Gastrointestinal	7
Ovary	8
Other tumor types	3

**Fig. 1:**
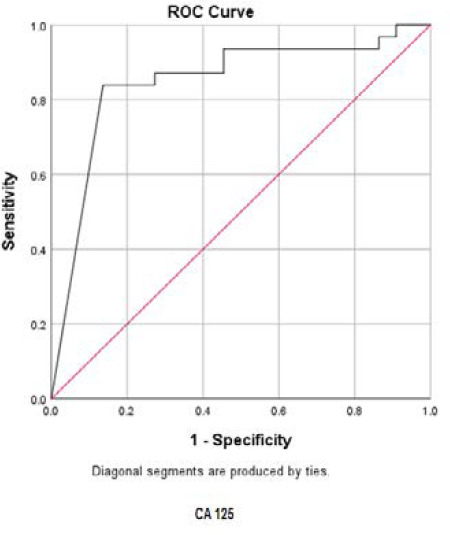
ROC curve for CA15-3

**Fig. 2:**
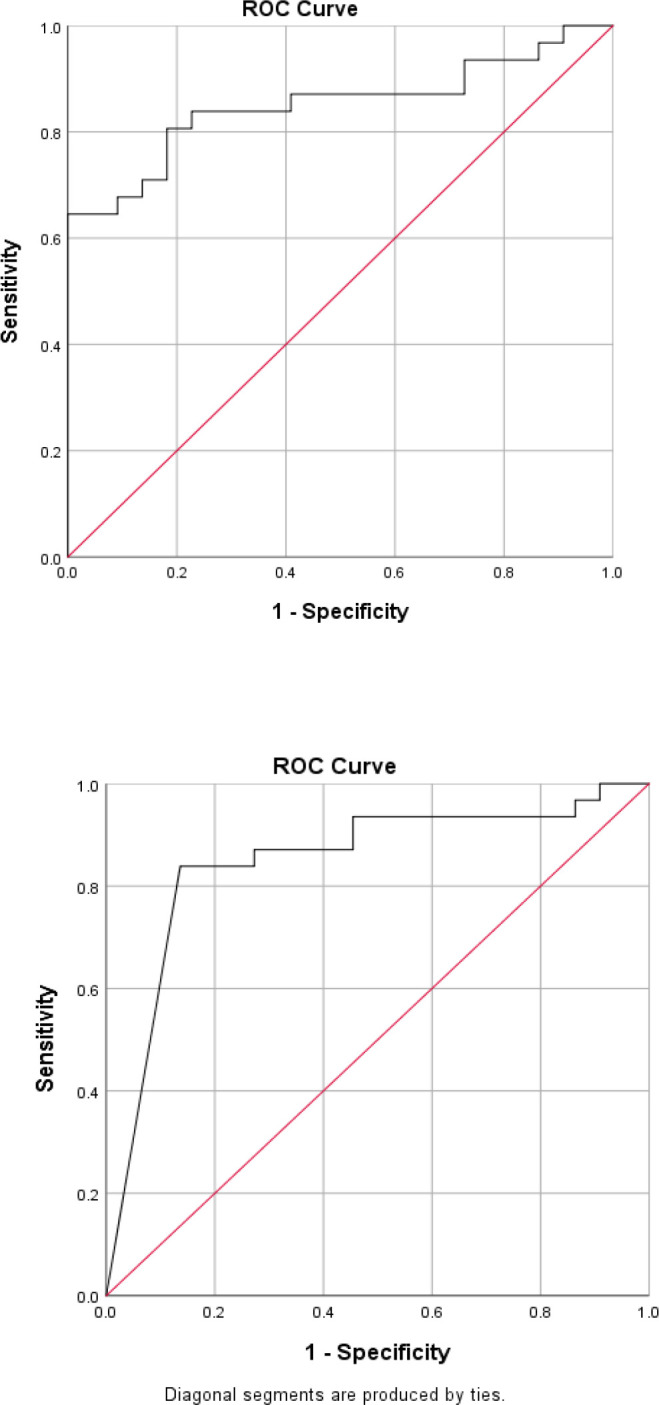
ROC curve for CA125

**Fig. 3:**
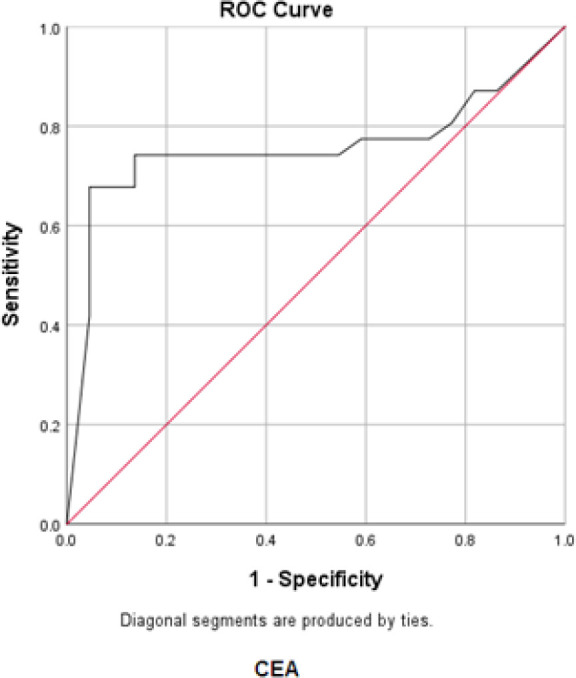
ROC curve for CEA

**Fig. 4:**
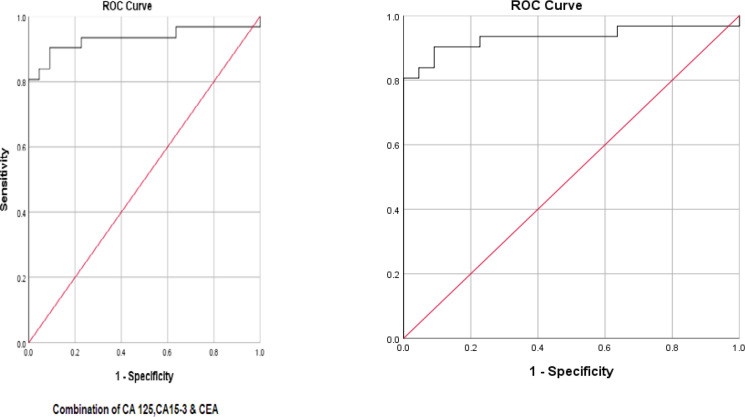
ROC curve for three tumor markers in combination

In order to determine the sensitivity and the specificity of the tumor markers levels in cancer diagnosis (positive cytological result), ROC curves were plotted for each tumor marker separately, as well as for a combination of all three tumor markers (Figures 1-4).

Accordingly, the best cut-off point and the sensitivity and specificity values were calculated for each tumor marker value (Table 4).

The best cut-off point is actually the number that has the highest accuracy in distinguishing cancer patients from those of non-cancer. These cut-off points were 39.55 u/mL for CA15-3, 486.00 u/mL for CA125, and 6.90 ng/mL for CEA. In the meantime, if we use the information on the combination of three complementary markers to predict the risk of cancer, this figure would be 0.38. The areas under the curve (AUC) for all three tumor markers consists of CA15-3, CA125 and CEA were 0.85 ± 0.054, 0.85 ± 0.058, and 0.75 ± 0.071, respectively. Also, if we use the information on the combination of three tumor markers to predict the risk of cancer, the AUC curve would be 0.93 ± 0.039. The closer this number to 1, the better the differentiation and accuracy power of the test will be. These results revealed that CA15-3, CA125, and CEA markers levels were respectively 85%, 85%, and 75%, higher in the randomly selected positive cytology (malignant) group as compared to the negative cytology (benign) group. The above probability for the combination of three tumor markers would be 93%. The most efficient predictive tumor marker is the one with the highest sensitivity and specificity value. As can be seen, all three tumor markers produced the same results; however, it seems that the combination of three tumor markers yields much more reliable predictive value.

According to the AUC criterion, one of the best criteria for determining the predictive power, the use of information on the combination of three tumor markers is the best method to predict the risk of cancer. The resulting cut-off point (0.38) for the combination of three tumor markers showed the highest specificity (91%) and sensitivity (90%). The sensitivity value indicated that 90% of the patients were diagnosed correctly, and the specificity value indicated that 91% of benign cases were diagnosed correctly. The closer the sensitivity and specificity values to 100, the higher the accuracy and differentiation power of the test will be (Table 5).

**Table 5:** Diagnostic value of single tumor marker and the combination of complementary triple markers

Tumor marker	The mean ± standard deviation of AUC	95% confidence interval	P-value	Cut-off point	Sensitivity (%)	Specificity (%)	Youden Index*	Positive Predictive Value (PPV)	Negative Predictive Value (NPV)
CA15-3	0.85±0.054	0.74-0.96	<0.001	39.50	64%	100%	64%	73.3%	85.7%
CA125	0.85±0.058	0.73-0.96	<0.001	486.00	84%	86%	70%	80.8%	75%
CEA	0.75±0.071	0.62-0.90	0.002	6.20	68%	96%	63%	67.7%	95.5%
CA15-3 + CA125 + CEA	0.93±0.039	0.86-0.1	<0.001	0.38	90%	91%	81%	87%	93.3%

*:Youden's J statistic (also called the Youden's index). The maximum distance between the ROC curve and the baseline model. PPV: positive predictive value. NPV: negative predictive value. AUC: area under the ROC curve

## Discussion

One of the less invasive methods for examination of the diffusion of pleural and abdominal serous fluids is fluid puncture (TAP). Conventional cytology is one of the crucial methods for examination of serosal fluid by which presence and origin of the malignancies are evaluated, however, it shows low clinical sensitivity for diagnosis of malignant lesions (approximately 60%) (2, 15).

The conventional cytology alone is associated with a low clinical sensitivity and is usually unable to determine the primary source of the malignancy in serosal fluids. The gold standard for specifying the type and origin of malignancies is studying of pleural biopsy, laparoscopy biopsy or open surgery.

There have been numerous studies aiming at improving none-invasive diagnostic methods, one of which is the study of biomarkers in serum serous fluids. 

Article reviews and meta-analysis of the pleural fluids of CEA, CA125, CA19-9, and CA15-3, showed that the combined use of the mentioned tumor markers along with morphology was helpful (16-18). However, poor sensitivity value was reported (5).

Purcell* et al.*, (2004) in a comprehensive study on 416 patients showed that malignant pleural fluid contains significantly higher levels of tumor markers in the effusion samples. The combination of four tumor markers (CEA>50 ng/mL, CA125>2800 u/mL, CA15-3>75 u/mL, and CYFRA 21.1>175 ng/mL) yielded a clinical sensitivity of 54%. They suggested that panel analysis of complementary markers could be used as an adjunct to the cytological diagnosis of malignancy (15).

In another study on 77 pleural fluid samples, Mehrabi* et al.*, (2005) reported the highest specificity value (100%) for a combination of serum CA15-3and CA15-3 ,CEA and NSE in pleural fluids and the highest sensitivity (80%) for a combination of serum CA15.3and CA15-3, NSE, and CEA in pleural fluids by ELISA method (19).

In a study on 74 serous fluid samples, Kornke* et al.*, (2009) showed that combination of two or more tumor markers could be effective in enhancing the diagnostic value. Overall, pleural fluid tumor markers outperformed the serum markers in detecting the etiology of pleural effusion (20).

Another meta-analysis done by Liang* et al.*, (2008) revealed the same results with the combination of complementary tumor markers, for example AUC for CEA/CA125 was reported as 0.86 and 0-0.93 for CEA/CYFRA 21-1in pleural fluid (21).

The application of complementary biomarkers may improve diagnostic accuracy. Ferrer* et al.*, (1999) achieved 65% sensitivity and 100% specificity for a combination of CEA, CA125, and CA15-3 assessment in a serosal fluid (22).

The literature review demonstrated that there were no independent tumor markers with excellent sensitivity and specificity value, as well as high diagnostic value in distinguishing between benign and malignant fluids.

Based on the earlier studies, a combined evaluation of complementary tumor markers is one of the methods that can increase the diagnostic value of biomarkers (19, 20).

In the present study, the CEA, CA125, and CA15-3 markers levels of 72 samples collected from three teaching hospitals in the categories of benign, suspicious, and malignant serous fluids (pleural fluid and ascites) were analyzed by 4^th^ generation Chemiluminescence immunoassay in Imam Hossein Core Laboratory.

According to the ROC statistical analysis, AUC achieved as much as 85%, 85%, and 75%, for CEA, CA125, and CA15-3 tumor markers, respectively; it means that 85-73% of patients randomly selected from the malignant group demonstrated significantly higher tumor markers than the benign group. 

The sensitivity values of these tumor markers were 64%, 84%, and 68% fpr CEA, CA125, and CA15-3respectively; also, the specificity was 100%, 86%, and 96%, respectively. 

In our analysis, AUC, sensitivity, and specificity of the combination of the above three tumor markers were calculated as 93%, 90%, and 91%, respectively. Some studies, such as the one carried out by Kernke* et al.*, showed the AUC of CEA, CA125, CYFRA 21-1, and NSE tumor markers was 89% (20).

Previous studies revealed lower diagnostic values for the serum measurement equal to 0.65. Overall, serosal fluid markers were superior to the serum markers in determining the pleural fluid etiology (20). However, no concomitant serum assay was performed in the present study. 

On the other hand, the serum specimen of our patients was not usually available in practical use in cytopathology laboratories. However, it was possible to assay complementary biomarkers along with cytomorphology on a single fluid specimen.

Results of the present study also showed that the diagnostic sensitivity of a single tumor marker, especially in a combination, was more than the clinical sensitivity of conventional cytology. The increased amount in the sensitivity and specificity of complementary tumor markers in the serosal fluid was 26% and 5%, respectively.

In the present study, the Youden indices for CEA, CA125, CA15-3, and the combination of these tumor markers were 63%, 70%, 64%, and 81%, respectively. The Youden index is a useful parameter enabling a comparative analysis of data found by various authors when single values of sensitivity and specificity are reported. The diagnostic yields of the three most accurate markers CEA, CA125, and CA15-3 calculated with the Youden index were virtually the same as those assessed with the AUC in our study.

We determined the best cut-off point as 39.55 u/mL for CA15-3, 486.00 u/mL for CA125, and 6.90 ng/mL for CEA in this immunoassay platform.

Furthermore, CA125 showed a higher correlation between morphology and markers based on the correlation coefficient analysis. According to the Spearman correlation coefficient, increase in tumor markers level leads to an increase in the diagnostic power of the cytological result. 

##  Conclusion

The present study demonstrated that non-invasive techniques such as complementary biomarkers assay with well-developed methods (4^th^ generations of immunoassay) in combination with conventional cytology may lead to an acceptable increase in the diagnostic power of fluid cytology which was in line with previous studies.

The advanced technology for ultra-sensitive detection of tumor markers can be used instead of conventional immunoassay methods (enzyme-linked immunosorbent assay and radio-immunoassay). The electrochemical devices are rapid, simple, and alternative methodologies for determination of the fluid tumor markers. The limitation of this study was that, the reference ranges and cut-off values of these markers differ in pleural and peritoneal fluids. It can be interpreted by different metabolism, half-life, and turn-over of the markers in the serosal fluids. On the other side, there are no unanimous cut-off points and reference ranges of these analyses in the body fluids that may be due to immunoassay heterogeneity and complexities. Many physiological conditions such as renal and hepatic functions can directly affect the levels of these biomarkers in fluids. In the future, multivariate statistical analysis, and novel immunoassay methods such as multiplex immunoassay, SERS-based multiplex immunoassay, and reverse phase protein array may significantly improve the diagnostic accuracy of fluid cytology examination for the comprehensive tumor marker panels.
